# Clinical application of chromosome microarray analysis and karyotyping in prenatal diagnosis in Northwest China

**DOI:** 10.3389/fgene.2024.1347942

**Published:** 2024-11-06

**Authors:** ShuYuan Xue, YuTong Liu, LiXia Wang, Le Zhang, Bozhen Chang, GuiFeng Ding, PengGao Dai

**Affiliations:** ^1^ The College of Life Sciences, Northwest University, Xi’an City, Shanxi, China; ^2^ Prenatal Diagnosis Center, Urumqi Maternal and Child Healthcare Hospital, Ürümqi City, Xinjiang Uygur Autonomous Region, China; ^3^ College of Public Health, Xinjiang Medical University, Ürümqi, Xinjiang Uygur Autonomous Region, China; ^4^ Department of Obstetrics, Urumqi Maternal and Child Healthcare Hospital, Ürümqi City, Xinjiang Uygur Autonomous Region, China

**Keywords:** karyotype, chromosomal microarray, prenatal diagnosis, ultrasonography, copy number variant

## Abstract

**Introduction:**

Karyotyping and chromosome microarray analysis (CMA) are the two main prenatal diagnostic techniques currently used for genetic testing. We aimed to evaluate the value of chromosomal karyotyping and CMA for different prenatal indications.

**Methods:**

A total of 2084 amniocentesis samples from pregnant women who underwent prenatal diagnosis from 16 to 22 + 6 weeks of gestation between January 2021 and December 2022 were retrospectively collected. The pregnant women were classified according to different prenatal diagnostic indications and underwent CMA and karyotype analysis. Clinical data were collected, and the results of the CMA and karyotype analysis were statistically analyzed to compare the effects of the two diagnostic techniques.

**Results:**

The total detection rate of abnormal chromosomes was significantly higher using CMA than karyotype analysis. The detection rate of abnormal chromosomes using CMA was significantly higher than that using karyotyping for ultrasound abnormalities, high-risk serologic screening, adverse pregnancy history, positive noninvasive prenatal test (NIPT) screening, and ultrasound abnormalities combined with adverse pregnancy history indications. Among the fetuses with inconsistent results between the two testing methods, 144 had an abnormal CMA but a normal karyotype, with the highest percentage of pregnant women with ultrasound abnormalities at 38.89% (56/144). CMA had the highest detection rate for structural abnormalities combined with soft-index abnormalities among all ultrasound abnormalities. The highest detection rate of copy number variants in the group of structural abnormalities in a single system was in the genitourinary system (3/29, 10.34%).

**Conclusion:**

CMA can improve the detection rate of chromosomal abnormalities in patients with ultrasound abnormalities, high-risk serologic screening, adverse maternal history, positive NIPT screening, and ultrasound abnormalities combined with adverse maternal history and can increase the detection rate of chromosomal abnormalities in karyotypic normality by 6.91% (144/2,084), this result is higher than similar studies. However, karyotype analysis remains advantageous over CMA regarding balanced chromosomal rearrangement and detection of low-level chimeras, and the combination of the two methods is more helpful in improving the detection rate of prenatal chromosomal abnormalities.

## 1 Introduction

The global prevalence of children with congenital structural or functional anomalies ranges between 5% and 7% (Slavotinek et al., 2023), with genetic factors contributing to more than 80% of birth defects (Lipinski and Krauss, 2023). A large number of genetic abnormalities during pregnancy may lead to miscarriages, and surviving infants may be born with a wide range of health complications, such as structural anomalies, developmental deficiencies, intellectual disabilities, and shortened life expectancy (McIntyre et al., 2021).

Karyotyping and chromosome microarray analysis (CMA) are the two main prenatal diagnostic techniques currently used for genetic testing. Karyotyping can be used to detect aneuploidy and large structural rearrangements (≥5–10 Mb) and has been the gold standard for the detection of abnormal chromosomes in prenatal diagnosis for the past few decades. However, it is limited in the detection of chromosomal microduplications and microdeletions, among others, at <5 Mb. CMA provides approximately 100-fold higher resolution than karyotyping and can detect aneuploidies, unbalanced rearrangements, triploid, uniparental disomy, and genome-wide copy number variants (CNVs) as small as 1–50 kb, which are associated with many known genetic syndromes or abnormalities. Therefore, CMA has been increasingly advocated as an optimal test for prenatal diagnostic testing for birth defects (American College of Obstetricians and Gynecologists’ Committee on Practice Bulletins—Obstetrics Committee on Genetics Society for Maternal-Fetal MedicineCommittee on GeneticsSociety for Maternal-Fetal Medicine, 2020; Kearney et al., 2011; Riggs et al., 2021). CMA has been shown to provide an additional 4%–6% diagnostic yield relative to karyotyping; however, the difference in detection rates between the two methods across different prenatal diagnostic indications has rarely been examined (Zhang J. et al., 2021), The wide variety of prenatal diagnostic indications and the variability in the types of chromosomal abnormalities associated with them affect detection rates, and it is unclear whether CMA is superior to karyotyping in all prenatal indications.

Therefore, this study retrospectively examined 2084 cases who underwent karyotyping and CMA testing for different prenatal diagnostic indications. Firstly, the detection rates of chromosomal abnormalities by the two methods were compared, and the differences in the detection rates of CMA and karyotyping under different prenatal diagnostic indications were investigated. Secondly, we provided specific genotype-phenotype associations in ultrasound-abnormal fetuses during follow-up, providing clinicians and high-risk pregnant women with valuable clinical data for their prenatal genetic counseling regimens.

## 2 Materials and methods

### 2.1 Patients and indications

This study was approved by the Ethics Committee of the Urumqi Maternal and Child Health Hospital and was conducted in accordance with the local legal and institutional requirements (XJFYLL2022015). Written informed consent was obtained from the pregnant women or their families for the publication of any potentially identifiable data included in this article.

**FIGURE 1:**
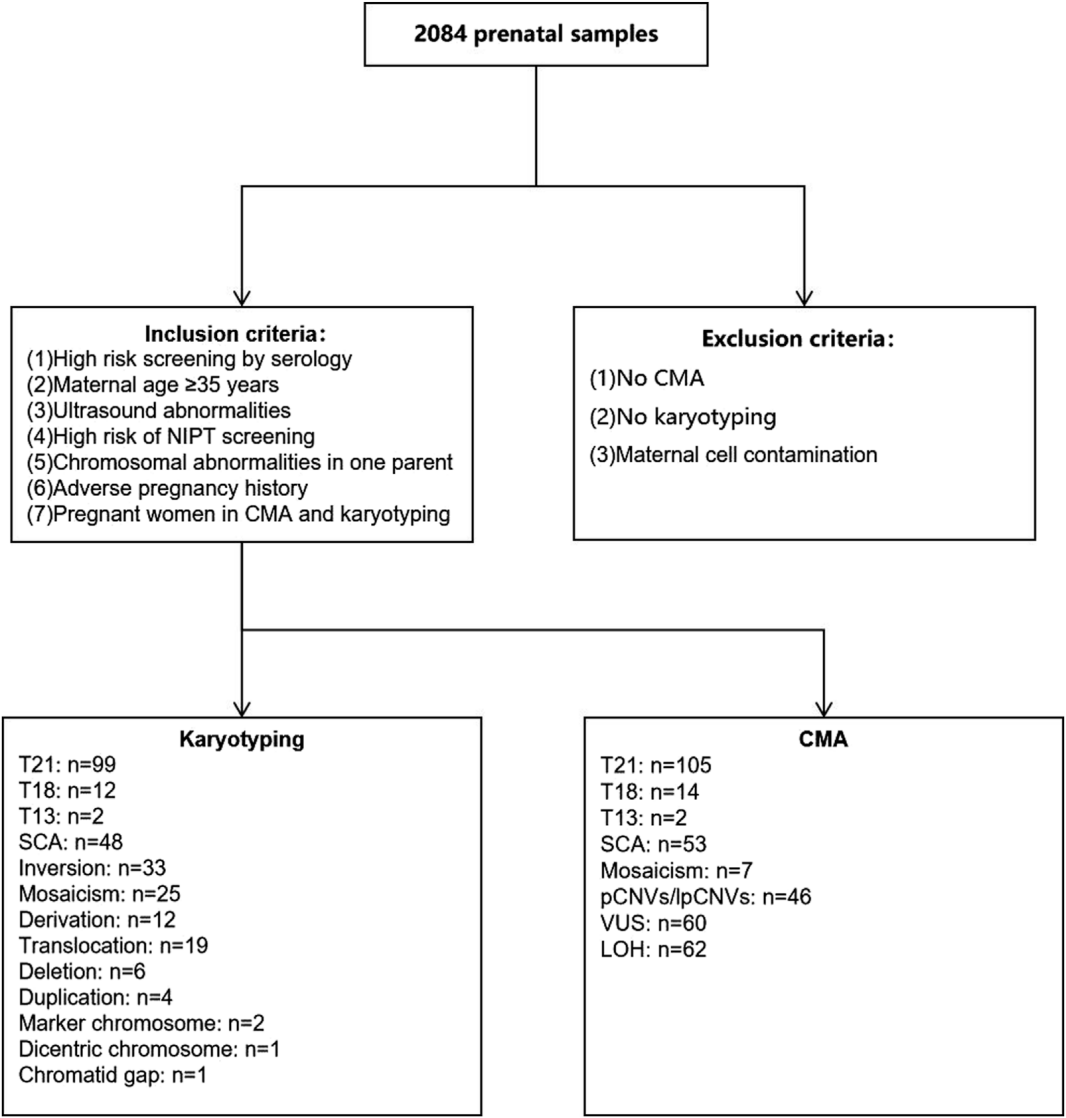
Flow diagram of inclusion and exclusion criteria as well as chromosomal results. CMA, chromosomal microarray analysis; T21, Trisomy 21; T18, Trisomy 18; T13, Trisomy 13.

A total of 2084 amniocentesis specimens were retrospectively collected from pregnant women who underwent prenatal diagnoses from 16 to 22 + 6 weeks of gestation at the Urumqi Maternal and Child Health Hospital between January 2021 and December 2022. The inclusion criteria were as follows: pregnant women with 1) high-risk serologic prenatal screening; 2) advanced maternal age (AMA, maternal age ≥35 years); 3) structural abnormalities on fetal ultrasound screening; 4) positive noninvasive prenatal test (NIPT) screening; 5) a history of adverse maternal outcomes: including miscarriage, preterm labour, etc.,; and 6) a history of chromosomal structural abnormalities or abnormal chromosome number syndromes in previous pregnancies. Pregnant women were categorized into one-, two-, or three-indication groups, according to the number of indications for prenatal diagnosis. A flowchart of the study is shown in Figure 1. The characteristics of the study population, including the indications for prenatal testing, are presented in Table 1.

**TABLE 1:** Characteristics of the study population.

Characteristic	Study population (*n* = 2084)
Maternal age	31 (28, 35]
Nation	
Han	1,436 (68.91%)
Uyghur	363 (17.42%)
Hui	120 (5.76%)
Kazakh	95 (4.56%)
Mongol	33 (1.58%)
Other	37 (1.78%)
Gestational age at invasive diagnosis (weeks)	21 (19.71, 23.86]
Indications for invasive prenatal diagnosis	
Single indication	
Ultrasound abnormalities	624 (29.94%)
High-risk screening by serology	421 (20.20%)
AMA	252 (12.09%)
High risk of NIPT screening	240 (11.52%)
Adverse pregnancy history	154 (7.39%)
Chromosomal abnormalities in one parent	15 (0.72%)
Two-indications	
AMA + Ultrasound abnormalities	89 (4.27%)
AMA + Adverse pregnancy history	83 (3.98%)
AMA + High risk of NIPT screening	55 (2.64%)
High risk of NIPT screening + Ultrasound abnormalities	43 (2.06%)
Ultrasound abnormalities + Adverse pregnancy history	35 (1.68%)
High risk of NIPT screening + Ultrasound abnormalities	27 (1.30%)
AMA + High risk of NIPT screening	14 (0.67%)
Chromosomal abnormalities in one parent + Adverse pregnancy history	12 (0.58%)
High risk of NIPT screening + Adverse pregnancy history	10 (0.48%)
Three-indications	
AMA + High risk of NIPT screening + Ultrasound abnormalities	10 (0.48%)

### 2.2 G-banding karyotyping

Amniotic fluid (20 mL) was obtained from pregnant women and collected in two equal portions in sterile tubes, one for G-banding karyotyping and one for CMA. Amniotic fluid (10 mL) was cultured using the wall culture method for 10–14 days. The growth of cells was observed daily. After 8–9 days of culture, cell growth was observed under an inverted microscope. When there were more than 12 adherent cell clones covering more than half of the field of view under a× 10 microscope, with many round translucent cells, 100 μL colchicine (final concentration 10 μg/mL) was added for 30 min and the cells were collected. The cells were harvested and routinely prepared, and G-banding was applied. Karyotype analysis was performed using a chromosome analyzer. The diagnostic standard of karyotype was based on the International System for the Nomenclature of Human Cytogenetics 2020 (ISCN 2020) for the karyotype analysis (McGowan-Jordan et al., 2020). Thirty cells were counted in each specimen, 4–5 karyotypes were analyzed, and 100 cells were counted for chromosomal chimerism.

### 2.3 Chromosomal microarray analysis

Amniotic fluid (10 mL) was obtained from pregnant women, and the genomic DNA was extracted with a DNA extraction kit (QIAamp DNA Blood Mini Kit) and stored at −70°C for testing. Five microliters of whole genomic DNA were digested with the NspI enzyme, then the adaptor was ligated. The sample DNA was amplified to 150–2,000 bp and purified using the magnetic bead method. The purified product was fragmented and labeled by fragmentase, and the whole genome Affymetrix Cytoscan 750 K Array microarray chip (Affymetrix Inc.) was used to amplify the DNA sample. High-abundance genomic DNA was obtained by PCR amplification using specific primers. The PCR product was purified by magnetic beads. After the concentration reached the standard, the DNA was fragmented using fragmentase, and the ends of the small fragments were labeled by terminal deoxynucleotide transferase. The labeled product was added to the Cytoscan 750 K Array microarray chip (Affymetrix, United States) for hybridization for 16–18 h, and the unhybridized DNA was removed by washing. The genome-wide Affy-metrix Cytoscan 750 K Array chip (Affymetrix) was used to detect clinically significant chromosomal microdeletions/microduplications, chromosomal subtelomeric deletion syndrome, other abnormal CNVs, and loss of heterozygosity (LOH).

### 2.4 Interpretation of chromosomal microarray analysis results

The pathogenicity of the detected CNVs was determined by comparison and analysis with public databases, such as DECIPHER, OMIM, ClinGen, DGV, and NCBI, and a literature search. The pathogenicity of CNVs was categorized into pathogenic CNVs (pCNVs), likely pathogenic CNVs (lpCNVs), variant of uncertain significance (VUS), likely benign CNVs (lbCNVs), and benign CNVs (bCNVs), according to the guidelines of the American College of Medical Genetics (Riggs et al., 2020). The dose effect, the number of genes and main pathogenic genes contained in the variation area, and the clinical significance of the variation were clearly demarcated: pathogenic (P) (≥0.99 score), likely pathogenic (LP) (0.98–0.90 score), variant of undetermined significance, VOUS) (0.89 to −0.89 points), likely benign (LB) (−0.90 to −0.98 points), and benign (benign, B) (≤-0.99 points). Results with conflicting variant classifications are first reviewed to ensure that there are no conflicting interpretations due to technical errors. Second, it can be combined with other testing techniques, such as FISH, MLPA and second-generation sequencing, to further validate conflicting test results. Finally, clinical information such as the patient’s clinical presentation and family history can be combined to determine which interpretation is more consistent with reality.

### 2.5 Statistical analysis

Data were analyzed using IBM SPSS Statistics version 24.0 (IBM Corp., Armonk, N.Y., United States). Non-normally distributed data were expressed as “median [lower quartile, upper quartile].” Categorical variables are represented by “n (%)” and analyzed using the chi-squared test. Statistical significance was set at *p* < 0.05.

### 2.6 Follow-up

Clinicians recorded clinical and pregnancy outcome data and obtained follow-up information by telephone until 1 year after the birth of the infant, during which lost cases were removed. Information on the fetus after birth was collected, including pregnancy outcomes, postnatal growth, physical examinations, major malformations, and other complications, or infant death.

## 3 Results

### 3.1 Basic clinical characteristics of 2,084 pregnant women

Overall, 2,084 patients underwent prenatal diagnosis with both CMA and karyotyping. They were categorized into 1,706 cases (81.86%) in the single-indication group, 368 cases (17.66%) in the two-indication group, and 10 cases (0.48%) in the three-indication group, according to different prenatal diagnosis indications. In the single-indication group, 624 patients had ultrasound abnormalities, 421 had high-risk serologic screening, 252 had AMA, 240 had NIPT-positivity, 154 had adverse maternal history, and 15 had a chromosomal abnormality in one of their parents. For additional details, see Table 1.

### 3.2 Comparison of detection rate between chromosome karyotype and microarray analysis

CMA detected a total of 349 cases of abnormal chromosomes, of which 239 cases were in the single-indication group, 104 in the two-indication group, and six in the three-indication group, with detection rates of 14.01%, 28.26%, and 60.00%, respectively. Chromosomal karyotyping and CMA analyses showed significantly higher detection rates in the two- and three-indication groups than in the single-indication group (*p* < 0.05). A total of 264 cases of abnormal chromosomes were detected by karyotype analysis, including 168 cases in the single-indication group, 90 in the two-indication group, and six in the three-indication group, with detection rates of 9.85%, 24.46%, and 60.00%, respectively.

**FIGURE 2:**
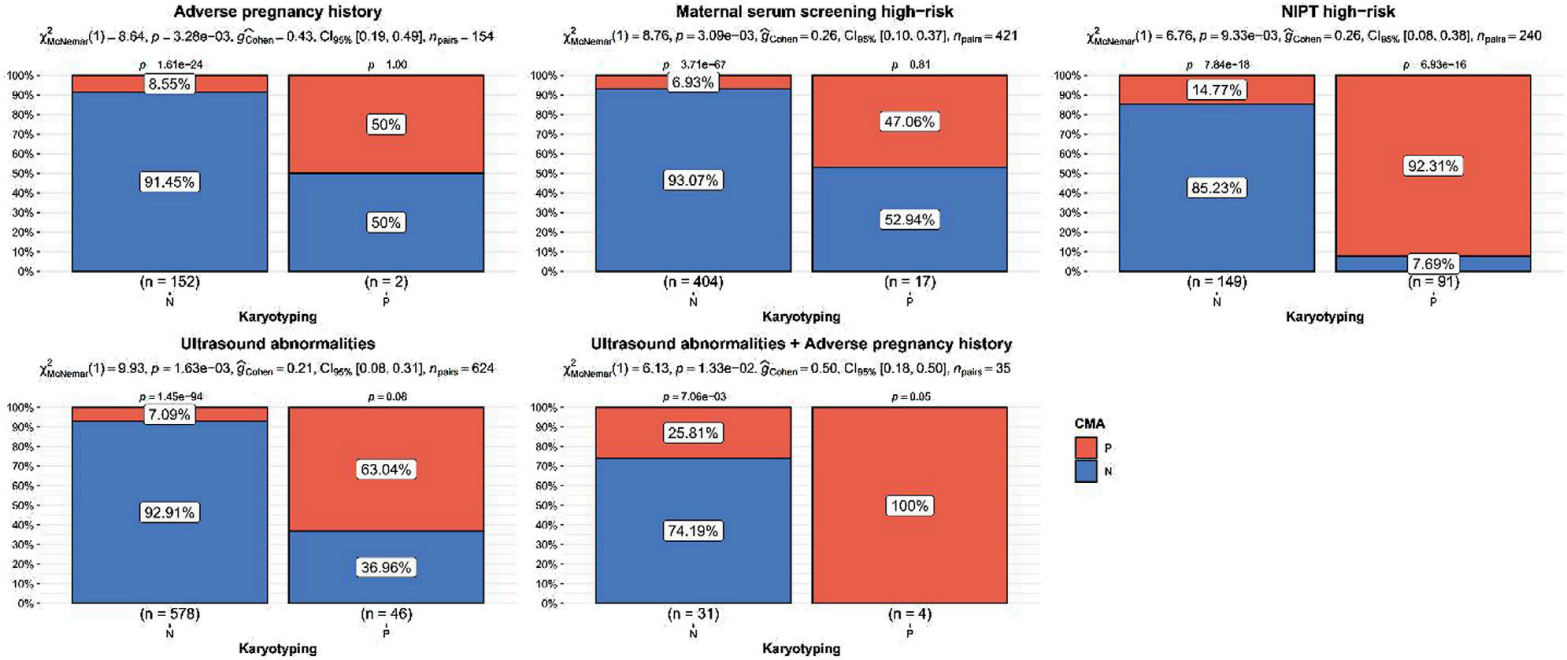
Indications of significant difference between chromosomal microarray analysis and karyotyping detection rate. *p* values were the statistical difference of constituent ratios by Chi-square test between karyotyping and CMA.

The detection rate of CMA was significantly higher than that of karyotyping for ultrasound abnormalities, high-risk serologic screening, adverse maternal history, and NIPT-positivity (*p* < 0.05). No statistically significant difference was observed between the detection rates of CMA and karyotyping for the other indications (*p* > 0.05) (Figure 2; Table 2). Comparing the results of CMA with karyotyping, CMA is more effective in identifying small CNVs that are difficult to detect by conventional karyotyping. These CNVs include but are not limited to, deletions or duplications of chromosome segments smaller than 5 Mb, low proportional chimerism, and complex chromosomal rearrangements.

**TABLE 2:** Abnormal karyotype/chip detection rates for different prenatal diagnostic indication subgroups.

	Indication for prenatal diagnosis	Number of cases (n)	Karyotype (%)	CMA (%)	*P*
Single indication					
	Ultrasound abnormalities	624	46 (7.37)	70 (11.22)	<0.01*
	Maternal serum screening high-risk	421	17 (4.04)	36 (8.55)	<0.01*
	AMA	252	8 (3.17)	11 (4.37)	
	NIPT high-risk	240	91 (37.92)	106 (44.17)	<0.01*
	Adverse pregnancy history	154	2 (1.30)	14 (9.09)	<0.01*
	Chromosomal abnormalities in one parent	15	4 (26.67)	2 (13.33)	
	Total	1,706	168 (9.85)	239 (14.01)	<0.001*
Two- indication					
	AMA + Ultrasound abnormalities	89	18 (20.22)	21 (23.60)	
	AMA + Adverse pregnancy history	83	4 (4.82)	3 (3.61)	
	AMA + NIPT high-risk	55	28 (50.91)	33 (60.00)	
	Maternal serum screening high risk + Ultrasound abnormalities	43	4 (9.30)	4 (9.30)	
	Ultrasound abnormalities + Adverse pregnancy history	35	4 (11.43)	12 (34.29)	<0.05*
	NIPT high-risk + Ultrasound abnormalities	27	24 (88.89)	25 (92.59)	
	AMA + Maternal serum screening high-risk	14	1 (7.14)	3 (21.43)	
	Chromosomal abnormalities in one parent + Adverse pregnancy history	12	6 (50.00)	0 (0.00)	
	Maternal serum screening high risk + Adverse pregnancy history	10	1 (10.00)	3 (30.00)	
	Total	368	90 (24.46)	104 (28.26)	<0.05*
Three-indication					
	AMA + NIPT high-risk + Ultrasound abnormalities	10	6 (60.00)	6 (60.00)	
	Total	10	6 (60.00)	6 (60.00)	
Total		2,084	264 (12.67)	349 (16.75)	<0.01*

CMA, chromosomal microarray analysis; AMA, advanced maternal age; *The difference between the detection rates of CMA, and conventional karyotype abnormalities was statistically significant, *p* < 0.05.

### 3.3 Inconsistent results between karyotype and chromosomal microarray analysis

In our study, a total of 349 cases of chromosomal abnormalities were detected by CMA, with the highest percentage of chromosome number abnormalities at 50.72% [177/349, detection rate: 8.49% (177/2,084)], pCNVs/lpCNVs at 13.18% [46/349, detection rate: 2.21% (45/2,084)], VUS at 17.19% [60/349, detection rate: 2.88% (60/2,084)], and LOH at 17.77% [62/349, detection rate: 2.98% (62/2,084)].

Karyotype analysis detected a total of 264 cases of chromosomal abnormalities, with chromosome number abnormality being the most common abnormality at 70.45% [186/264, detection rate: 8.93% (186/2,084)]. Other abnormalities included chromosomal inversions, translocations, derivations, deletions, duplications, markers, double-stranded chromosomes, and chromosomal monosome gaps, as shown in Figure 1.

**FIGURE 3:**
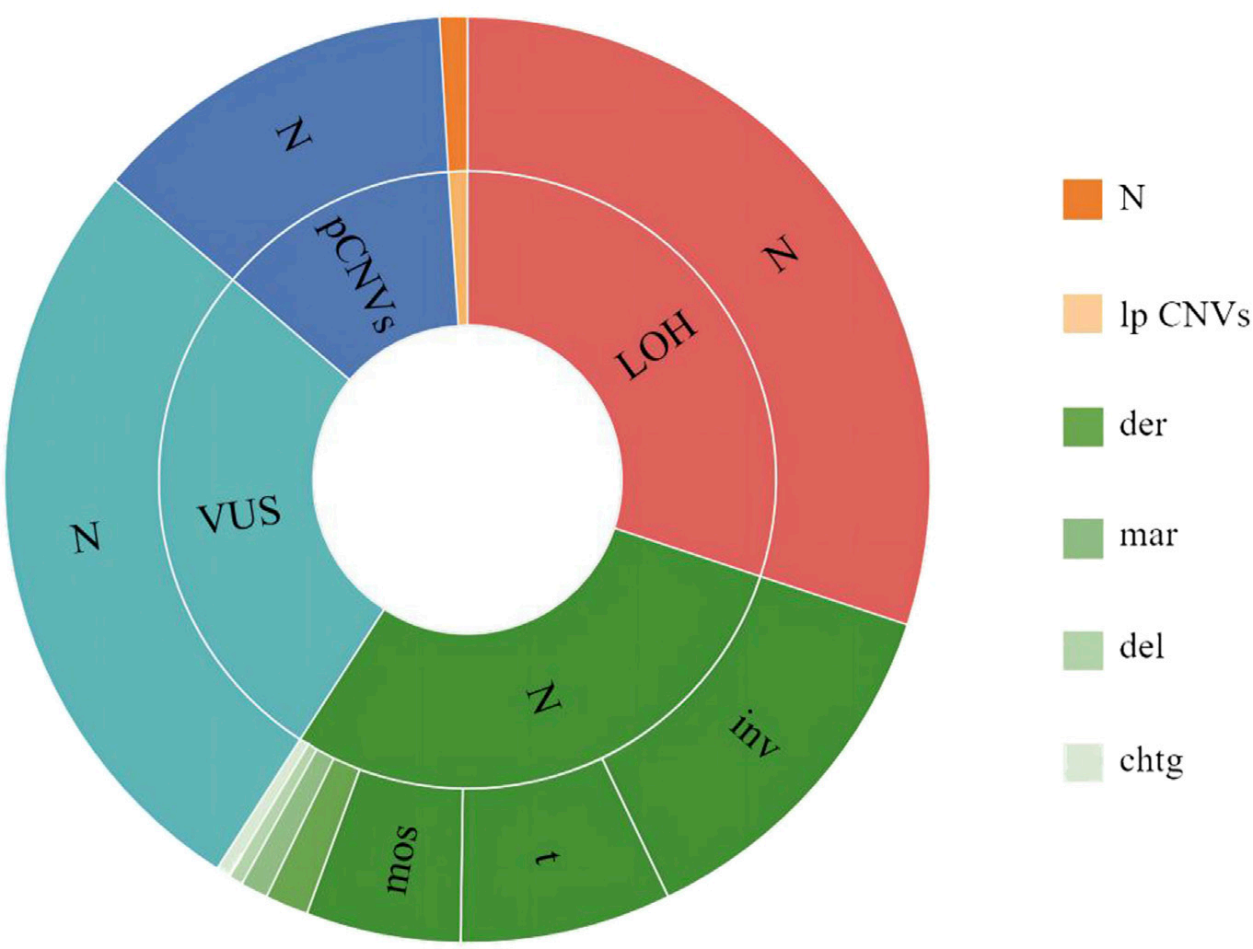
Inconsistent results between karyotyping and CMA. The outer circle indicates karyotyping results, and the inner circle indicates CMA results. N, normal; inv, inversion; t, translocation; mos, mosaicism; der, derivative chromosome; mar, marker chromosome; del, deletion; chtg, chromatid gap; LOH, Loss of Heterozygosity; VUS, variant of uncertain significance; pCNVs, pathogenic copy number variants; lpCNVs, likely pathogenic copy number variants.

Among the 2,084 samples, 144 had abnormal CMA but a normal karyotype, of which 61 had LOH, 55 had VUS, 28 were pathogenic/likely pathogenic; and 59 had an abnormal karyotype but normal CMA, of which 26 had chromosomal inversions, 15 had chromosomal translocations, 11 had mosaicism, three had derivative chromosome, two had marker chromosomes, one had a chromosomal deletion, and one had a chromatid gap. The highest percentage of pregnant women with ultrasonographic abnormalities among the 144 cases with abnormal CMA but a normal karyotype was 38.89% (56/144), as shown in Figure 3.

Therefore, 828 cases with fetal malformations detected by ultrasonographic screening were further analyzed and divided into five groups according to the ultrasound results: single structural abnormality, multiple structural abnormalities, structural abnormality combined with soft-index abnormality, single soft-index abnormality, and multiple soft-index abnormalities. Chromosomal abnormalities were analyzed in each group.

### 3.4 Chromosomal microarray analysis in fetuses with ultrasound abnormalities

A total of 828 pregnant women had fetal abnormalities on ultrasonography, and 138 cases (138/828, 16.67%) of chromosomal abnormalities were detected using CMA, including 71 cases (71/828, 8.94%) of chromosomal numerical abnormalities, 17 cases of LOH (17/828, 2.05%), and 49 cases of CNVs (49/828, 5.92%), among which 26 cases (26/828, 3.14%) had clinical significance.

The 828 specimens with ultrasound abnormalities were categorized based on their ultrasound characteristics into single-system structural abnormalities, multiple soft-index abnormalities, single soft-index abnormalities, structural abnormalities combined with soft-index abnormalities, and other abnormalities. The most common abnormality was a single soft-index abnormality in 472 patients (472/828, 57%), followed by multiple soft-index abnormalities in 148 patients (148/828, 17.87%), other abnormalities in 121 patients (121/828, 14.61%), structural abnormalities in a single system in 61 patients (61/828, 7.37%), and structural abnormalities combined with soft-index abnormalities in 26 patients (26/828, 5.41%).

The total chromosomal abnormality detection rates were as follows: structural abnormalities combined with soft-index abnormalities (13/26, 50.00%), multiple soft-index abnormalities (39/148, 26.35%), single soft-index abnormality (74/472, 15.68%), and single-system structural abnormality (9/61, 14.75%). The detection rates of clinically significant CNVs were as follows: structural abnormalities combined with soft-index abnormalities (7/26, 26.92%), single-system structural abnormalities (7/61, 11.48%), single-system soft-index abnormalities (26/472, 5.51%), and multisystem structural abnormalities (8/148, 5.41%), as shown in Table 3.

**TABLE 3:** Chromosomal microarray analysis in fetuses with ultrasound abnormalities.

Types of ultrasonic abnormalities	Number of people	Total chromosomal abnormalities (%)	Chromosomal numerical abnormality (%)	CNVs (%)	CNVs (%)		LOH (%)
					pCNVs/lpCNVs	VUS	
Single-system structural anomalies	61	9 (14.75)	1 (1.64)	7 (11.48)	4 (6.56)	3 (4.92)	1 (1.64)
Respiratory	4	1 (25.00)	0 (0.00)	1 (25.00)	1 (25.00)	0 (0.00)	0 (0.00)
Urinary system	29	4 (13.79)	0 (0.00)	3 (10.34)	1 (3.45)	2 (6.90)	1 (3.45)
Digestive system	6	1 (16.67)	1 (16.67)	0 (0.00)	0 (0.00)	0 (0.00)	0 (0.00)
Facial features	3	2 (66.67)	0 (0.00)	2 (66.67)	1 (33.33)	1 (33.33)	0 (0.00)
Skeletal limbs	19	1 (5.26)	0 (0.00)	1 (5.26)	1 (5.26)	0 (0.00)	0 (0.00)
Multiple soft indicator anomalies	148	39 (26.35)	24 (16.22)	8 (5.41)	4 (2.70)	4 (2.70)	7 (4.73)
Abnormalities in single soft indicators	472	74 (15.68)	40 (8.47)	26 (5.51)	14 (2.97)	12 (2.54)	8 (1.69)
NT/NF	136	24 (17.65)	14 (10.29)	8 (5.88)	1 (0.74)	7 (5.15)	2 (1.47)
NB	102	19 (18.63)	15 (14.71)	3 (2.94)	3 (2.94)	0 (0.00)	1 (0.98)
Choroid plexus cyst	23	4 (17.39)	3 (13.04)	1 (4.35)	0 (0.00)	1 (4.35)	0 (0.00)
Separation of renal pelvis	27	5 (18.52)	0 (0.00)	5 (18.52)	3 (11.11)	2 (7.41)	0 (0.00)
Intracardiac echogenic foci	34	6 (17.65)	3 (8.82)	2 (5.88)	2 (5.88)	0 (0.00)	1 (2.94)
Widening of the lateral ventricles	65	6 (9.23)	1 (1.54)	3 (4.62)	2 (3.08)	1 (1.54)	2 (3.08)
Echogenic enhancement of the intestinal tract	11	2 (18.18)	0 (0.00)	1 (9.09)	1 (9.09)	0 (0.00)	1 (9.09)
Subclavian vagus	25	1 (4.00)	0 (0.00)	1 (4.00)	1 (4.00)	0 (0.00)	0 (0.00)
Single umbilical artery	35	4 (11.43)	3 (8.57)	0 (0.00)	0 (0.00)	0 (0.00)	1 (2.86)
Femoral shortening	6	1 (16.67)	0 (0.00)	1 (16.67)	1 (16.67)	0 (0.00)	0 (0.00)
Widening of the posterior cranial fossa pool	8	2 (25.00)	1 (12.50)	1 (12.50)	0 (0.00)	1 (12.50)	0 (0.00)
Structural abnormalities combined with soft-index abnormalities	26	13 (50.00)	5 (23.08)	7 (26.92)	3 (11.54)	4 (15.38)	1 (3.85)
Others	121	3 (2.48)	1 (0.83)	1 (0.83)	1 (0.83)	0 (0.00)	1 (0.83)
Total	828	138 (16.67)	71 (8.57)	49 (5.92)	26 (3.14)	23 (2.78)	18 (2.17)

P, pathogenic; LP, likely pathogenic; VUS, variant of uncertain significance.

### 3.5 Follow-up results of ultrasound abnormalities detected by CMA only

Among the 828 patients with abnormal ultrasound results, 56 had normal karyotype results with chromosomal abnormalities detected using CMA. Among them, 19 cases were pCNVs/lpCNVs, 37 cases were of VUS. Among the 19 cases, 11 cases chose to terminate the pregnancy, five cases resulted in good fetal health, and three cases miscarried during pregnancy; among the 37 cases, 25 cases resulted in good fetal health, six cases chose to terminate the pregnancy, one case was born with cardiac anomalies, and five cases were lost to follow-up. The causes of termination of pregnancy (TOP) in patients included chromosomal aberrations, ultrasonographic abnormalities, miscarriage, preterm labor, and stillbirth.

## 4 Discussion

In this study, 2084 pregnant women in Xinjiang with prenatal diagnostic indications underwent karyotyping and CMA and were categorized according to the number of prenatal indications. The detection rate of abnormal chromosomes in the two- and three-indication groups was significantly higher than that in the single-indication group (*p* < 0.05). This indicates a greater likelihood of fetal chromosomal abnormalities with more prenatal diagnostic indications. Therefore, we suggest that that interventional prenatal diagnosis should be promptly performed with the presence of two or more prenatal indications, which can significantly increase the detection rate of pathogenic chromosomal abnormalities and reduce the risk of birth defects. In addition, in karyotypically normal amniotic fluid samples, CMA increased the detection rate of chromosomal abnormalities by 6.91%, which was higher than in similar studies (Hillman et al., 2013).

The results of our study showed that the overall detection rate of CMA abnormalities was 16.75%, which was 1.70% higher than the 12%–15% reported in postnatal studies of children with congenital anomalies, developmental delays, or intellectual disability (Armengol et al., 2012; Breman et al., 2012; Van Opstal et al., 2015; Chai et al., 2019; Rodriguez-Revenga et al., 2020). Furthermore, the detection rate of clinically significant CNVs in our study was 2.16%, which was slightly lower than the 2.59%–2.70% reported in previous multicenter studies (Levy and Burnside, 2019). In this study, the detection rate of chromosomal abnormalities using CMA was 4.08% higher than that of chromosomal karyotyping (*p* < 0.05), and karyotyping was limited to detecting whole-chromosome aneuploidy, large deletions, and duplications (≥5–10 Mb), polyploidy, and some balanced chromosomal rearrangements. However, CMA cannot completely replace karyotyping in prenatal diagnosis. Compared to CMA, karyotyping is a more traditional cytogenetic method that typically provides lower resolution but can reveal large-scale structural rearrangements such as inversions and translocations. CMA focuses on the detection of CNVs at a much higher resolution, using specific hybridization between probes and genomic DNA and fluorescent signal detection to identify genomic CNVs. Coupling the ability of karyotypes to detect large-scale structural anomalies with the high-resolution CNV detection of CMA provides a more comprehensive view of the genome than is possible with a single technology. Although CMA is a highly sensitive and specific technique for identifying CNVs in the genome, it has inherent limitations in the detection of certain types of chromosomal abnormalities. In particular, CMA is unable to detect balanced chromosomal rearrangements, such as balanced translocations, inversions, and insertions. These abnormalities involve the exchange of genetic material between chromosomes or within a chromosome but without a net gain or loss of genetic material. As a result, the total amount of DNA remains unchanged, making the changes undetectable by CMA, which relies on the detection of changes in DNA copy number.

CMA testing for balanced chromosomal rearrangements can have a significant impact on patient care decisions. First, balanced rearrangements can be associated with genetic disorders, including developmental delay, intellectual disability, and congenital malformations (Dardas et al., 2024). Their absence from CMA results may therefore lead to missed or delayed diagnoses, potentially affecting the timeliness and effectiveness of patient management. Second, balanced rearrangements may have reproductive health implications. If parents carry balanced translocations, they are at increased risk of passing on unbalanced rearrangements to their offspring, which can have serious health consequences. Identifying these carriers by karyotyping makes it possible to detect balanced rearrangements, allowing for appropriate genetic counseling and reproductive planning. In prenatal diagnosis, if a patient chooses CMA alone without traditional karyotyping, certain abnormalities in chromosome structure, such as balanced translocations, inversions, etc., may not be detected and a small percentage of chimeras may be missed.

The prenatal diagnostic rates of karyotyping and CMA for pregnancies with different indications varied widely, with some studies reporting that CMA increased the detection rate of chromosomal abnormalities in karyotypically normal fetuses by 6%–7% when fetal abnormalities were detected on prenatal ultrasonography, furthermore, this rate was approximately 1.7% for all other indications (Wapner et al., 2012). In the present study, the detection rate of chromosomal abnormalities was significantly higher (*p* < 0.05) in the cases of ultrasound abnormalities, high-risk serologic screening, poor maternal history, and NIPT-positivity, and the detection rate of chromosomal abnormalities using CMA was significantly higher than that using karyotyping (*p* < 0.05). An increasing proportion of fetal structural anomalies associated with genetic disorders have been reported in the literature (Borrell et al., 2023). The risk of chromosomal abnormalities is further increased if multiple soft index abnormalities or structural abnormalities are detected on ultrasound. This cumulative effect leads to a higher detection rate. In prenatal diagnoses, when structural abnormalities and soft-index abnormalities are found in the fetus, the possibility of chromosomal abnormalities should be highly suspected. This finding helps clinicians to more accurately assess the risk to the fetus and thus provide more rational genetic counseling and intervention to the pregnant woman. Studies have shown that testing with CMA in the presence of ultrasound abnormalities significantly improves the diagnosis of genetic disorders and contributes to improved clinical management and patient prognosis (Kim et al., 2023). The potential biological mechanisms are mainly based on the principle of dose-response of key genes. Specifically, an increase or decrease in the copy number of a gene will directly lead to a corresponding change in the expression of its encoded product, which in turn may disrupt the homeostasis of the intracellular environment, affecting the normal physiological function and developmental trajectory of the cell. In addition, more complex forms of structural variants, such as translocations, may disrupt the original expression control mechanism, and the disruption of this control mechanism may lead to abnormal gene expression or silencing, which may ultimately become potential triggers for the development of a variety of diseases. Therefore, the study of CNVs and structural variants not only helps to understand the complex relationship between genes and disease but also provides new perspectives and strategies for disease prevention, diagnosis and treatment.

Currently, CMA is recommended as the preferred detection modality for the prenatal diagnosis of fetal ultrasound structural abnormalities (American College of Obstetricians and Gynecologists Committee on Genetics, 2013). The probability of detecting clinically significant CNVs in 828 fetuses with ultrasound abnormalities in this study was 2.06%, which was generally consistent with the 2.60% previously reported in the literature (Lee et al., 2012). The American College of Obstetrics and Gynecology (ACOG) proposed the combination of chromosomal karyotyping and CMA when the ultrasound suggests the existence of structural abnormalities in the fetus (Author Anonymous, 2021), which can improve the detection rate of pCNVs. A major advantage of CMA is its ability to detect CNVs at the microscopic and submicroscopic levels that are difficult to identify by karyotyping, which can involve the amplification or deletion of single genes or small gene fragments with important consequences for gene expression and cellular function. In addition to CNVs, CMA can detect complex structural abnormalities such as chimeric conditions, where two or more genetically distinct cell lines are present in an individual, which is very common in certain genetic diseases. In addition, CMA can detect complex rearrangements involving multiple chromosomes, regions of homozygosity, variants that may be missed by karyotyping due to technical limitations. In this study, ultrasound abnormalities were detected in a total of 14 cases with normal chromosomal karyotypes, but pathogenic/possibly pathogenic results were obtained using CMA. The highest percentage (53.85%) further proved that the CMA plays an important role as a prenatal diagnostic guide for ultrasound abnormalities, suggesting that CMA testing is preferred for fetuses with ultrasound structural abnormalities.

Our study showed that the total chromosomal abnormality rate in the CMA abnormality group (44.17%) was higher than the detection rate of karyotypic chromosomal abnormalities (37.50%) in the NIPT-positivity group, which is consistent with previous studies (Zhu et al., 2021). The ability of NIPT to detect abnormalities in trisomy 21, trisomy 18, and sex chromosome numbers is excellent; nevertheless, CMA detects clinically significant CNVs, in addition to the diagnosis of those found to be at high risk of CNV syndrome by NIPT. All pregnant women with abnormal NIPT results are recommended to undergo CMA testing. In this study, the detection rate of abnormal karyotypes in AMA was 3.17%, and the total chromosomal abnormality rate detected by CMA was 4.37%, with no statistically significant difference. The results of this study showed that most CNVs detected in AMA were VUS, and there was no obvious age-related trend in CNVs, which is consistent with the results of previous studies (Srebniak et al., 2018). The total chromosomal abnormality rate in the abnormal serological screening group analyzed by CMA was 8.55%, and most of the abnormal chromosomes were LOH and VUS; furthermore, the detection rate was higher than that of the karyotyping. The detection rate of abnormal chromosomes by CMA in the high-risk serological screening group was 8.55%, and most of them were LOH and VUS; similarly, the detection rate was higher than that of the karyotyping. Therefore, it is recommended that pregnant women with high-risk serological screening are recommended to undergo combined karyotype and CMA testing.

Our analysis of 828 cases of maternal ultrasound abnormalities in this study showed that the rate of chromosomal abnormalities was significantly higher in those with structural abnormalities combined with soft-index abnormalities than in those with multiple soft-index abnormalities (*p* < 0.05), and the rate of chromosomal abnormalities in those with multiple soft-index abnormalities was significantly higher than that in those with a single soft-index abnormality (*p* < 0.05). Therefore, we recommend CMA testing in clinical practice for patients with structural abnormalities combined with soft-index abnormalities and for those with multiple soft-index abnormalities. In the subgroup of single-system structural abnormalities, the highest detection rate of CNV abnormalities was found in the genitourinary system (3/29, 10.34%). CMA has become a first-line tool for the effective diagnosis of patients with renal-related disorders (Groopman et al., 2018), and 4%–10% of postnatal patients with congenital renal and urinary tract anomalies carry pathogenic CNVs (Shaffer et al., 2012; Li et al., 2019; Su et al., 2022). In the subgroup of individual ultrasound soft-index abnormalities, nuchal translucency (NT) or nuchal fold (NF) thickening (136/472, 28.81%) and Nasal bone (NB) absence (102/472, 21.61%) were the most common abnormalities, which agrees with previous studies (Zhang Y. et al., 2021). Cases of CNVs with abnormal chromosome numbers and clinically significant CNVs were all more concentrated in two types of soft indicators: NT or NF thickening and NB deletion. The detection rate of clinically significant CNVs was higher when these soft indicators were combined with other ultrasound abnormalities. Ultrasound and chromosome results are essential for the early identification of genetic factors that may affect fetal development and outcome, and these results provide parents with direct information about the health status of their fetus. Through genetic counseling, parents can gain a fuller understanding of their fetus and make more informed decisions, as well as obtain an important reference point for future birth planning and family health planning.

We conducted a telephone-mode follow-up workup for patients with normal karyotype results among those with ultrasound abnormalities but abnormal CMA test results. Pathogenic/likely pathogenic CNVs are most commonly located on chromosomes 15, 16, 17, and 22. In this study, the 22q11.21 region was the most frequently detected. The presence of a high number of segmental duplications or low-copy repeats (LCR) on these chromosomes leads to non-allelic homologous recombination during meiosis, causing duplications and deletions of the corresponding segments (Ou et al., 2008). Among them, cases 8, 10, 11, and 13 had approximately 3 mb of duplications or deletions in the LCR22-A to LCR22-D region, which contains 49 OMIM genes, such as TBX1, CLTCL1, and HIRA, with clinical phenotypes including congenital heart disease, facial anomalies, and palatopharyngeal insufficiency, all of which were pathogenic CNVs, and the pregnancies were terminated after genetic counseling. Case 23 was a 735 kb copy number deletion that was confirmed by the parents to have originated from the father, who did not have an abnormal clinical phenotype, and the neonate’s growth and development showed no significant abnormalities at follow-up. Therefore, the fragment size and the origin of CNVs in the same region have a significant impact on the outcome of the pregnancy, and the consultant should make a comprehensive assessment based on family lineage information. One was induced after prenatal genetic counseling, and the other was miscarried during the pregnancy (Table 4). The range of the 17q12 microdeletion was 1.06–2.46 Mb, and HNF1B was the main causative gene, which was inherited in an autosomal dominant manner. The 17q12 deletion was initially found to be associated with adult-onset diabetes of young 5 (MODY 5), which was subsequently recognized as the third most common genetic disorder affecting the kidneys. As reported by Zhang Z. et al. (2021), the 17q12 microdeletion was associated with enhanced renal echogenicity, which was also combined with cardiac abnormalities in case 6, which is consistent with the literature (Mefford, 2016). In contrast, the ultrasound manifestation of bilateral renal pelvic separation in case 1 has not been reported previously, and the finding of this phenotype broadens the phenotypic spectrum of the 17q12 deletion. Unfortunately, in both cases, no further parental verification was done to clarify the source of the variant, which in such cases is an important guide for the family’s next pregnancy. In conclusion, CMA has become an indispensable diagnostic tool for a wide range of common microdeletion/microduplication syndromes, often associated with severe clinical phenotypes, because of its high efficiency and precision. The use of this technology also provides a solid basis for implementing timely and effective interventions and improving the overall quality of the population.

**TABLE 4:** Follow-up results of 56 cases.

Case	Ultrasound findings	CNVs	Chromosome band	Copy number	Pathogenicity	Pregnancy outcomes
1	Bilateral renal pelvis separation	17q12	1.5 Mb	Loss	P	TOP
2	Nasal bone dysplasia	17p12	1.4 Mb	Loss	P	BH
3	Left ventricular strong light spot	xp21.2p21.1	97 kb	Loss	P	TOP
4	cleft lip and palate	22q11.23	1.35 Mb	Gain	P	TOP
5	BPD 73 mm, FL 49 mm	1q21.1q21.2	4.7 Mb	Gain	P	TOP
6	Enhanced echogenicity of both kidneys, tricuspid regurgitation	17q12	1.48 Mb	Loss	P	MC
7	Nasal bone dysplasia	17p12	1.43 Mb	Loss	P	BH
8	echogenic bowel	22q11.21	2.3 Mb	Gain	P	TOP
9	Nasal bone dysplasia	16p11.2	599 Kb	Loss	P	TOP
10	Right aortic arches, left subclavian vagus	22q11.21	3.15 Mb	Loss	P	TOP
11	Right subclavian vagus; excessive amniotic fluid	22q11.21	3.1 Mb	Loss	P	TOP
12	Butterfly vertebra at Lumbar(L)5 level with widening of both renal pelvis	16p11.2	712 kb	Loss	P	TOP
13	NT: 4.6 mm; Left ventricular strong light spot	22q11.21	3.008 Mb	Gain	P	TOP
14	Unilateral widening of the lateral ventricles	17q12	1.485 Mb	Loss	P	MC
15	Fetal foot varus	Xp21.2p21.1	113 kb	Loss	LP	BH
16	widening of the lateral ventricles	2q37.3 14q32.33	2 Mb and 3.1 Mb 1.7 Mb	Gain and Loss Gain	LP VUS	TOP
17	Separation of both renal pelvises	4q35.2 16p11.2	2.6 Mb 777 kb	Loss Gain	VUS P	BH
18	Fetal heart structure abnormalities	Xp22.31	1.68 Mb	Gain	VUS	BH
19	Bilateral widening of the lateral ventricles	xp22.33p22.32	2.69 Mb	Loss	VUS	LTF
20	cleft lip and palate	10q11.22q11.23	4.1 Mb	Gain	VUS	TOP
21	Rocking chair foot, gallbladder enlargement	17q23.2q23.3	3.9 Mb	Gain	VUS	TOP
22	HL 39 mm, FL 44 mm	6p21.1	1.3 Mb	Loss	VUS	BH
23	Right renal absence	22q11.21	735 kb	Loss	VUS	BH
24	Fetal foot varus, Bilateral widening of the lateral ventricles	xp22.31	1.68 Mb	Loss	VUS	TOP
25	NT ≥ 3.0 mm (NT: 3.0 mm)	3q11.2	1.7 Mb	Loss	VUS	BH
26	NT ≥ 3.0 mm (NT: 3.6 mm)	9p24.2	1.54 Mb	Gain	VUS	BH
27	Posterior fossa cisterna widened	Xp22.31	1.68 Mb	Gain	VUS	BH
28	Choroid plexus cysts	xp22.33	985 kb	Gain	VUS	BH
29	separation of renal pelvis	15q12q13.1	1.59 Mb	Loss	VUS	BH
30	kidney fusion, echogenic bowel	13q33.1q33.3	3.89 Mb	Loss	VUS	TOP
31	NT ≥ 3.0 mm (NT: 3.2 mm)	20p11.23	1.23 Mb	Gain	VUS	TOP
32	Left ectopic pelvic kidney	Xp22.33	753 kb	Gain	VUS	BH
33	Left ventricular strong light spot, biparietal diameter and femur small for 3 weeks	5p13.3p13.2	1.96 Mb	Loss	VUS	LTF
34	NT ≥ 3.0 mm (NT: 3.3 mm)	Xp22.33	1.51 Mb	Gain	VUS	TOP
35	Single umbilical artery; Butterfly vertebra at Thoracic 11 level	22q13.31q13.33	2.1 Mb	Loss	VUS	BH
36	Nasal bone dysplasia; choroid plexus cysts	Yp11.2	3.0 Mb	Loss	VUS	BH
37	NT ≥ 3.0 mm (NT: 3.4 mm)	Xp22.31	1.68 Mb	Gain	VUS	BH
38	NT ≥ 3.0 mm (NT: 4.4 mm)	4p15.33	2.34 Mb	Loss	VUS	BH
39	Osteogenesis imperfecta of one side of the nasal bone	1 LOH	11.84 Mb		VUS	LTF
40	single umbilical artery	3 LOH	10.02 Mb		VUS	BH
41	Persistent left superior vena cava	3 LOH	17.3 Mb		VUS	BH
42	Bilateral renal pelvis separation, gallbladder enlargement, Aberrant right subclavian artery	4 LOH	11.31 Mb		VUS	BH
43	unilateral widening of the lateral ventricles	4 LOH	15.31 Mb		VUS	LTF
44	unilateral widening of the lateral ventricles and Posterior fossa cisterna widened	6 LOH	23.17 Mb		P	MC
45	NT ≥ 3.0 mm (NT: 3.1 mm)	12 LOH	15.85 Mb		VUS	LTF
46	Echogenic bowel	14 LOH	11.40 Mb		VUS	BH
47	Bilateral widening of the lateral ventricles	17 LOH	20.98 Mb		VUS	BH
48	NT ≥ 3.0 mm (NT: 3.4 mm)	19 LOH	10.70 Mb		VUS	BH
49	Ventricular septal defect, bilateral widening of the lateral ventricles	6,7 LOH	43.97/40.79 Mb		P	BH
50	Choroid plexus cysts	1,2,10 LOH	15.49/14.15/14.22 Mb		VUS	Cardiac Abnormality
51	Choroid plexus cysts; gallbladder enlargement	1,6,11 LOH	25.07/20.06/18.88 Mb		VUS	BH
52	lymphatic cyst	1,11,12,17 LOH	25.61/16.35/10.99/30.40 Mb		VUS	BH
53	Single umbilical artery with tortuous ductus arteriosus	2,9,13,16 LOH	34.93/10.77/13.19/20.46/27.70 Mb		VUS	BH
54	Polycystic kidney	3,6,7,8,18 LOH	15.15/22.39/41.03/15.14/12.23 Mb		VUS	BH
55	Cardiac anomalies; widening of lateral ventricles; posterior fossa cyst	X,3,5ROH	35.81/34.15/24.96 Mb		VUS	BH
56	Left ventricular strong light spot, echogenic bowel, NT ≥ 3.0 mm (NT: 3.3 mm)	X,9,10LOH	73.28/25.95/23.34 Mb		VUS	BH

CMA, chromosomal microarray analysis; Del, deletion; Dup, duplication; NT, nuchal translucency.

TOP, pregnancy termination; BH, born healthy; MC, miscarriage; LTF, Lost to follow-up.

P, pathogenic; LP, likely pathogenic; VUS, variant of uncertain significance.

This study has some limitations, including a retrospective design and the presence of strong sample selectivity. Furthermore, the CMA test results in our study were mostly of unknown clinical significance. The follow-up system can be enhanced in the future by combining advanced data analytics and machine learning algorithms to predict potential complications and adjust interventions accordingly to monitor patient outcomes. In addition, we propose a prospective study with a larger and more diverse sample to validate the results of our current study. This would include recruiting participants from different geographical locations and demographics to ensure the generalizability of our findings.

## Data Availability

The datasets presented in this study can be found in the ClinVar repository (https://www.ncbi.nlm.nih.gov/clinvar/). The accession number(s) can be found in the Supplementary Material.
